# Effects of coated cysteamine hydrochloride on muscle fiber characteristics and amino acid composition of finishing pigs

**DOI:** 10.5713/ajas.18.0414

**Published:** 2018-10-26

**Authors:** Miaomiao Bai, Hongnan Liu, Kang Xu, Rong Yu, Abimbola Oladele Oso, Jinping Deng, Yulong Yin

**Affiliations:** 1 Scientific Observing and Experimental Station of Animal Nutrition and Feed Science in South-Central, Ministry of Agriculture, Hunan Provincial Engineering Research Center for Healthy Breeding of Livestock and Poultry, Key Laboratory of Agro-ecological Processes in Subtropical Region, National Engineering Laboratory for Pollution Control and Waste Utilization in Livestock and Poultry Production, Institute of Subtropical Agriculture, Chinese Academy of Sciences, Changsha 410125, China; 2 College of Animal Science, South China Agricultural University, Guangzhou, Guangdong 510642, China; 3 Hangzhou King Techina Technology Company Academician Expert Workstation, Hangzhou King Techina Technology Co., Ltd., Hangzhou 311107, China; 4 Hunan Co-Innovation Center of Animal Production Safety, CICAPS, Changsha, Hunan 410128, China; 5 Department of Animal Nutrition, College of Animal Science and Livestock Production, Federal University of Agriculture, Abeokuta PMB 2240, Nigeria

**Keywords:** Coated Cysteamine Hydrochloride, Finishing Pigs, Muscle Fiber, Amino Acids

## Abstract

**Objective:**

This experiment was designed to determine the effects of coated cysteamine hydrochloride (CC) on muscle fiber characteristics, amino acid composition and transporters gene expression in the *longissimus dorsi* muscle (LDM) of finishing pigs.

**Methods:**

Two hundred and sixteen Duroc/Landrace/Yorkshire cross-bred male finishing pigs were fed with a corn-soybean basal diet supplemented with 0, 70, and 140 mg/kg cysteamine. Each group contained eight replicates of nine pigs per replicate. After 29 days, one pig was randomly selected from each replicate and slaughtered. Blood and LDM samples were collected and analyzed.

**Results:**

The results showed that supplemental dietary CC increased (p<0.05) the muscle fiber density. And CC supplementation also up-regulated (p<0.05) the expression of myosin heavy chain 1 (MyHC1) and MyHC2x mRNA levels, and down-regulated (p<0.05) MyHC2b expression in the LDM. Additionally, supplemental dietary CC reduced (p<0.05) the concentration of total cholesterol in the plasma and enhanced (p<0.05) the concentrations of essential amino acid and total amino acid in the LDM. The relative expression levels of chloramphenicol acetyltransferase 2, b^0,+^ amino acid transporter, and y^+^-L-type amino acid transporter 1 were up-regulated (p<0.05) in the LDM when pigs were fed with the dietary CC of 70 mg/kg.

**Conclusion:**

Cysteamine supplementation could increase fiber density and distribution of fiber types. It also improved the deposition of protein in the LDM by up-regulated the expression of amino acid transporters.

## INTRODUCTION

Meat quality is an important economic trait in meat industry, with the freshness and nutritional value of meat being the key factors that governs consumers’ buying decision [1]. However, with the intensive selection for greater growth rate and lean percentage of pigs, meat quality also has deteriorated [2]. Therefore, identifying new ways to improve meat quality and nutritive value is important for human health and animal productivity. Meat quality is affected by the interaction between many factors such as muscle structure, postmortem environmental conditions, meat processing, packaging, and storage conditions, etc. [3]. Muscle fiber characteristics are the basic and direct factors to affect meat quality [4]. Muscle fiber composition is received to distinguish red and white muscles, which predicts biochemical changes and consequently meat quality [5].

Meat protein value has been assessed according to its amino acids composition. The amino acid composition is considered as an important index for nutritional value of each food. Numerous studies have verified that dietary supplementation with extra amino acids can influence the amino acid profile of tissues, particularly muscles [6]. Additionally, different amino acids are transported by specific amino acid transporters (neutral, acidic, or basic) in the muscle. The difference of quantitative expression of amino acid transports reflects the process of amino acid metabolism.

Cysteamine, as a novel feed additive in animal production, is used in the form of coated cysteamine hydrochloride (CC). Numerous researches have investigated its biological activity, which induces the regulation of mTOR signaling for modifying protein metabolism and antioxidative activities [6,7]. Using the commercial pig model, the previous study has shown supplemental dietary cysteamine improved the stability of pork color by regulating oxidation [8]. Cysteamine is a physiological performance promoter, which regulates protein synthesis and protein degradation in pigs [9]. The influence of cysteamine supplementation on protein digestion and absorption may be due to its improving jejunal amino acid transportation [10]. However, the effects of cysteamine on the regulation of amino acids metabolism in the muscle has not been investigated. The ability of cysteamine to enhance the muscle fiber characteristics of finishing pigs remains unexplored. A coating product prevents its dissolution or disintegration in the gastric environment. Therefore, based on these previous studies, it was hypothesized that supplementation with CC might influence muscle fiber characteristics, muscular deposition of protein and gene expression of transporters in the *longissimus dorsi* muscle (LDM) of finishing pigs thereby improving its meat quality and nutritive.

## MATERIALS AND METHODS

### Animals and sampling

The animal experiment protocol (NO. 20160712) was approved by the animal welfare committee of the Institute of Subtropical Agriculture, The Chinese Academy of Sciences, Changsha, China.

Two hundred and sixteen cross-bred (Duroc/Landrace/Yorkshire) castrated pigs with an average initial weight of 88.3±0.3 kg were used in this study. Pigs were individually divided into three treatment groups, and each group was replicated eight times with nine pigs per replicate. The basal corn-soybean meal diet was respectively supplemented with 0 (control, CON), 70 mg/kg (CC70), and 140 mg/kg (CC140) cysteamine supplied by coated CC (basal dietary composition is presented in Table 1). The basal diet was formulated to meet the nutritional requirement of finishing pigs recommended by the NRC [11]. Pigs had free access to feeds and drinking-water throughout the experimental stage. The experiment lasted for 29 d. Additionally, coated CC, supplied by Hangzhou King Techina Technology Co., Ltd (Hangzhou, China), contained 270 g/kg CC.

After a 29-day period feeding experiment, one pig randomly selected from each pen was fasted overnight and the morning for 12 h and the killed by exsanguination after electrical stunning (250 V, 0.5 A, for 5 to 6 s). Fresh blood samples from external jugular vein were collected and kept in vacuum tubes with ethylenediaminetetraacetic acid. Plasma was obtained by centrifugation at 3,500×g for 15 minutes at 4°C, and the supernatant stored at −20°C for analyzing biochemical indices. Muscle samples were carefully removed from the LDM at between 6th and 7th thoracic vertebrae within 45 min postmortem. The LDM samples were manually trimmed into 0.5×0.5×1.0 cm pieces, promptly fixed in 10% formalin for microsection manufacture. Subsequently, about 20 g LDM samples were rapidly excised and weighted, then freeze-dried for amino acid analysis. Moreover, the extra LDM sample was packaged in foil, promptly frozen in liquid nitrogen, and stored at −80°C for molecular analysis.

### Plasma biochemical indices

Plasmas were centrifuged at 3,500×g for 15 minutes at 4°C, and the supernatants were used to determine biochemical indices by an Automatic Biochemical Analyzer (F. Hoffmann-La Roche Ltd, Basel, Switzerland). These plasma biochemical indices, containing the concentrations of total protein, triglyceride, total cholesterol, glucose, urea nitrogen, and the activities of aspartate aminotransferase, alanine aminotransferase, and alkaline phosphatase, were analyzed according to the commercial reagents form Roche Diagnostics GmbH (Roche Diagnostics GmbH, Mannheim, Germany) and manufacturer’s instructions [12].

### Muscle fiber characteristics measurement

Muscle fiber density and diameter were measured by the traditional staining of hematoxylin and eosin (HE). Samples of the LDM were transferred form 4% paraformaldehyde to 70% ethanol. Before dehydrated through a serial alcohol gradient, individual sections of muscle biopsy material were placed in processing cassettes. The sections embedded into paraffin wax blocks were cut into 5μm thickness and dewaxed in xylene, then rehydrated through lower concentrations of ethanol. After washed in phosphate buffer saline, sections were stained with HE and dehydrated through increasing concentrations of ethanol and xylene. The morphology of the LDM was observed at 200 times under a light microscope [13]. The Image-pro plus-6.0 pathologica system was applied for imaging analysis, and three views were selected for each section. Muscle fiber characteristics were determined by measuring muscle fiber density and diameter. Approximately 10 views were counted to estimate the cross-sectional area of the muscle fiber. Muscle fiber density was expressed as the ratio of total number of fibers counted to the total area of muscle fiber measured. Muscle fiber diameters were analyzed using the Image-Pro plus 5.1, which was an image analysis system (Media Cybernetics Inc., Rockville, MD, USA) [14].

### Amino acid profile in muscle

Amino acid compositions (hydrolytic amino acids) of the LDM were determined by an amino acid analyzer (L8900, Hitachi, Tokyo, Japan). Amino acids were extracted from tissue following the technique described by Hu et al [15] with slight modifications. About 0.1 g of freeze-dried muscle samples was hydrolyzed with 10 mL of hydrochloric acid solution (HCl, 6 M) in a sealed ampoule bottle for 22 h at 110°C. Then all hydrolysate was diluted with double-distilled water in volumetric flask of 100 mL, subsequently 1 mL of hydrolysate diluent was passed through a 0.22 mm membrane filter. Samples were analyzed for amino acids contents through comparing peak profiles of the squid samples with standard amino acid profiles.

### Real-time quantitative polymerase chain reaction analysis

Total RNA was extracted from LDM samples using a Trizol Reagent (Invitrogen, Carlsbad, CA, USA) following manufacturer’s instructions and dissolved in diethylpyrocarbonate (DEPC)-treated water. The extracted RNA was quantified by measuring optical density at 260 and 280 nm using an Eppendorf Biophotometer (Eppendorf AG, Hamburg, Germany) and its integrity verified by electrophoresis on a 1% agarose gel. The cDNA was reverse-transcribed from 1 μg of total RNA using the DNase I treatment (Takara, Otsu, Japan), then diluted and used for evaluating gene expression. All primer sequences employed herein were developed previously for amplification of mRNA sequences of *Sus scrofa* (Table 2). These target genes respectively includes muscle fiber types (myosin heavy chain 1 [MyHC-1], 2a, 2b, and 2x), and amino acid transporters (autologous stem cell transplantion 2 [ASCT2], chloramphenicol acetyltransferase [CAT]-1 and 2, y^+^-L-type amino acid transporter [y^+^LAT1], LAT2, and b^0,+^ amino acid transporter [b^0,+^AT]). Amplification of the housekeeping gene (glyceraldehyde-3-phosphate dehydrogenase) and target genes was performed in a 10-μL reaction volume including 1 μM of each forward and reverse primer, 2 μL of cDNA, 2 μL of DEPC-treated water, and 5 μL of SYBR Premix Ex Taq (Takara Bio Inc., Japan). The relative expression levels of genes were performed using Lightcycler-480II system (Roche Diagnostics GmbH, Germany). The real-time polymerase chain reaction (RT-PCR) protocol was strictly carried out with forty cycles of amplification, with each cycle consisting of pre-denaturation at 95°C for 10 s; denaturation at 95°C for 5 s; annealing at 60°C for 30 s; and elongation at 52°C to 60°C for 30 s, followed by a melting curve analysis. Cycle threshold (Ct) values were employed to determine expression levels of genes and calculated of triplicate measurements. The mRNA expression levels were analyzed and expressed as the relative values to those for control pigs. The comparative Ct value method was descried by Tan et al [16].

### Statistical analysis

The experimental data were analyzed statistically by the one-way analysis of variance method using SAS (SAS Institute Inc., Cary, NC, USA; 2002) and expressed as means±standard error of the mean. Based on adjusted degrees of freedom solution, this data about muscle fiber characteristics measurements and amino acid composition were calculated using mixed procedure for repeated measures. Duncan’s multiple-range test was performed for indicating differences between significant mean values. The statistical differences were declared significant at p<0.05 in all analyses.

## RESULTS

### Plasma biochemical indices

Plasma biochemical indices in finishing pigs were tested and shown in Table 3. CC70 markedly reduced (p<0.05) the concentration of total cholesterol in the plasma when compared with the control and CC140 groups. However, compared with CC140 group, supplemental dietary 70 mg/kg CC significantly reduced (p<0.05) alanine aminotransferase activity in the plasma.

### Muscle fiber characteristics

**Figure 1:**
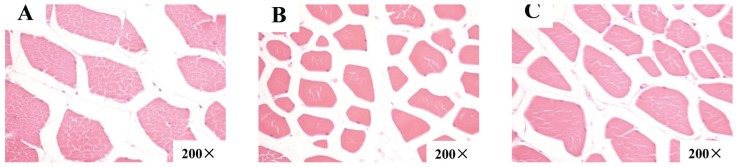
Morphology of *longissimus dorsi* muscle (LDM) fiber (200×). (A) Morphology of LDM fiber in pigs fed a basal diet containing 0 (CON). (B) Morphology of LDM fiber in pigs fed a basal diet containing 70 mg/kg (CC70). (C) Morphology of LDM fiber in pigs fed a basal diet containing 140 mg/kg (CC140).

The morphology of LDM fiber is shown in Figure 1. Based on fiber counting methods, the muscle fiber characteristics of finishing pigs in each group were measured and the data were analyzed, as shown in Table 4. In the same field of vision, there were more muscle fibers in the LDM in pigs fed the CC70 diet. Compared to control treatment and CC140, CC70 had a higher muscle fiber density (p<0.05) in the LDM.

### Gene expression levels of myosin heavy chain

**Figure 2:**
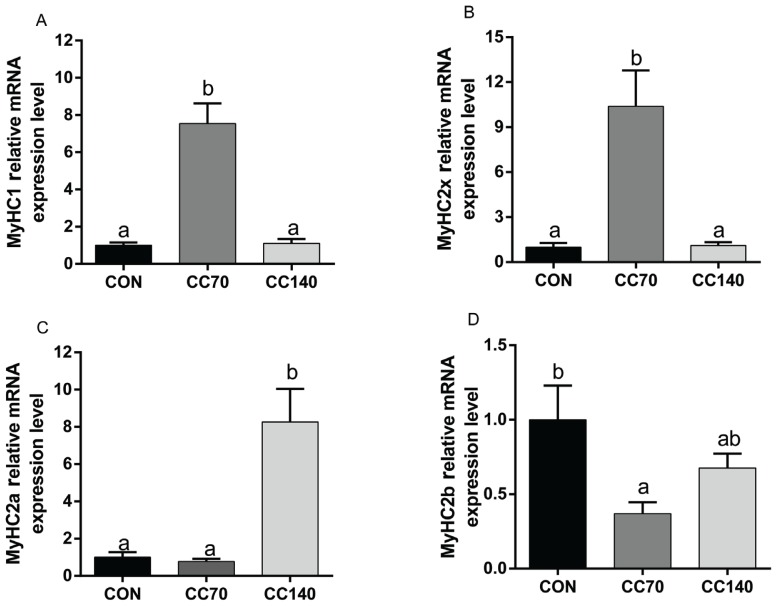
Expression levels of muscle fiber types of finishing pigs fed a basal diet containing 0 (CON), 70 (CC70) and 140 mg/kg (CC140) coated cysteamine hydrochloride, respectively. The figure indicates: (A) MyHC1; (B) MyHC2x; (C) MyHC2a; (D) MyHC2b. The error bars represents standard error. Mean values with unlike letters (a, b) were significantly different (p<0.05).

As shown in Figure 2, effects of dietary CC supplementation on the mRNA level of MyHC isoform gene of finishing pigs were assessed via RT-PCR. Supplemental dietary 70 mg/kg CC significantly up-regulated (p<0.05) muscle MyHC1 and MyHC2x mRNA levels. Meanwhile, compared to control group, a decreased muscle MyHC2b mRNA level (p<0.05) was observed with dietary 70 mg/kg CC supplementation. And the expression level of MyHC2a was lower (p<0.05) in pigs fed control and CC70 than CC140 diet.

### Amino acid profile in muscle

The amino acid profile that indicates protein metabolism in the LDM is detailed in Table 5. The concentrations of essential amino acid and total amino acid (TAA) were calculated based on the analyzed muscle amino acid contents in this study. Compared with CC140, supplemental dietary 70 mg/kg CC markedly improved (p<0.05) the muscle TAA. Leucine, isoleucine and valine are known as branched-chain amino acids. A significant increased isoleucine (p<0.05) was observed with pigs fed CC70 diet. Moreover, compared to control group, CC70 significantly enhanced (p<0.05) the concentration of histidine in the LDM. In the neutral amino acids, CC70 increased (p< 0.05) the content of phenylalanine. Dietary 70 mg/kg CC supplemented pigs had higher (p<0.05) asparagine content in the LDM than control and 140 mg/kg CC.

### Gene expression levels of amino acid transporters

**Figure 3:**
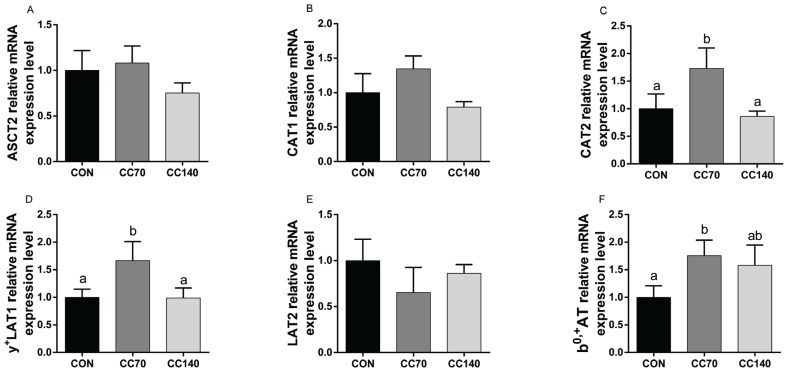
Effects of dietary coated cysteamine hydrochloride (CC) on the relative mRNA expression levels of ASC amino-acid transporter 2 (ASCT2), cationic amino acid transporter (CAT)-1, CAT2, L-type amino acid transporter (LAT)-2, y^+^LAT1, and b^0,+^ amino acid transporter (b^0,+^AT) in (A, B, C, D, E and F) the longissimus dorsi muscle of finishing pigs. The figure indicates which pigs were fed with a corn-soybean diet containing the CCH of 0 (CON), 70 (CC70), and 140 mg/kg (CC140), respectively. The error bars represents standard error. Mean values with unlike letters (a, b) were significantly different (p<0.05).

To further explore the different influences of dietary CC on muscle amino acid composition, we measured the expression of key amino acid transporters The relative mRNA expression levels of CAT2, y^+^LAT1 and b^0,+^AT were up-regulated (p<0.05) by 70 mg/kg CC supplementation of the diet compared to the control diet (Figure 3C, 3D, 3F). However, no significant difference (p>0.10) was observed on ASCT2, CAT1, and LAT2 expression in the LDM between groups (Figure 3A, 3B, 3E).

## DISCUSSION

In livestock production dietary supplementation with certain nutrients is supposed to be an effective way of improving meat quality and nutrition [17]. Numbers of studies *in vivo* and *in vitro* have focused on the growth-promoting and antioxidant functions of cysteamine [18,19]. However, the previous studies have demonstrated that supplemental dietary CC had no significant effect on meat pH and drip loss, but increased meat color values and the deoxymyoglobin content by improving glutathione levels and antioxidant activity [20]. Meanwhile, this present study is first to indicate that dietary cysteamine supplementation can improve meat quality of finishing pigs through changing muscle fiber density and type distribution and promoting amino acids deposition and transportation.

Muscle fiber, as an important component of muscle, directly affects meat quality. Muscle fiber characteristics have been correlated with various meat quality traits including muscle pH, tenderness, drip loss, meat color, and intramuscular fat content [21]. Moreover, muscle fiber number, size and fiber type composition are closely interrelated and were implicated in muscle fiber characteristics [22]. The fiber density was positively related to tenderness of cooked pork [23,24]. In the present study, dietary CC supplementation increased muscle fiber density in finishing pigs which indicates improving the sensory quality of meat.

Additionally, muscle fiber types can determine the size, num ber and density of muscle fiber. In general, muscle fibers are divided into four types considered as slow-oxidative type I, fast oxido-glycolytic type IIA, and fast glycolytic types IIX and IIB, and expressed by MyHC isoforms genes identified as MyHC1, MyHC2a, MyHC2b, and MyHC2x [25]. According to Kim et al [26], the content of myoglobin and fiber color increases in the rank order I>IIA>IIX>IIB, but the fiber size is in the opposite order. A higher type 1 fiber proportion might be positively correlated with muscle fiber density. Previous researches also reported that low percentages of type 2A and 2B fibers in the meat resulted in increased lightness (L*) and redness (a*). This is agreed with our previous study, which suggested that pigs fed dietary CC had increased a* in the LDM which promoted the stability of pork color [19]. This was related to the myoglobin content of different fiber types. Furthermore, it has been demonstrated that antioxidant status is positively correlated with a* or color stability in the LDM of beef [27]. Cysteamine, as an excellent scavenger of oxidants hydroxyl radical and hypochlorous acid, can protect against body oxidation. A muscle fiber type transition toward more oxidative muscle fibers such as MyHC1 can be induced by antioxidant [28]. This present study firstly provides the results that dietary CC supplementation improves MyHC1 and MyHC2x expression and decreases MyHC2b mRNA expression, which may partly explain the mechanism of cysteamine on the improvement of meat quality.

Intramuscular amino acid composition and metabolism are determined to evaluate the biological value of muscle protein nutrition contributing to meat quality. This present study indicated that dietary CC supplementation enhances the muscle amino acids deposition of finishing pigs. Consistent with previous studies, cysteamine supplementation resulted in a marked stimulation of protein deposition in skeletal muscle [6,29]. Importantly, plasma metabolism profile is reflected by changes in physiological and metabolic activities in response to dietary manipulations [30]. In the present study, serum total cholesterol was decreased, and glucose was increased by dietary supplemented with CC, indicating the inhibition of lipid metabolism and acceleration of glucose metabolism. Cysteamine could accelerate glucose and amino acid into muscle for protein synthesis, which may be associated with an increase in insulin-like growth factor 1 consequently stimulating protein synthesis [31].

Additionally, specific transporters plays an important role for transporting amino acids into the cell and in response to amino acid availability [32]. Previous studies have found that amino acid transporters changed in small intestine, skeletal muscle, and other tissues once dietary nutrient content was varied [33]. At present, little information about the impact of cysteamine on amino acid transporters in the LDM is available. However, the branched-chain amino acids (Ile), alkaline amino acids (His) and neutral amino acids (Phe and Ans) contents were increased in the LDM in the present study. Different amino acid transporters unquestionably play a critical role in transportation with different mechanisms and featuring with unique substrate specificities [34]. The cationic amino acid transporters (CAT) have an important role in the transportation of arginine, lysine, histidine, and ornithine to regulate their homeostasis, and are widely distributed in tissues [35]. The ASCT2, b^0,+^AT, y^+^LAT1, and LAT2 are neutral amino acids transporters, and are mainly involved in the transport of branched-chain amino acids and some small neutral amino acids including asparagine and glutamine [36]. In addition, b^0,+^AT, and y^+^LAT1 are Na^+^-independent transporters [37]. This agreed with Zhou et al [10], who reported that cysteamine supplementation in finishing pigs enhanced protein digestion and absorption via upregulating the expression of amino acid transporters (y^+^LAT1 and b^0,+^AT) in the jejunum. Thus, it is possible that there is an inevitable correlation between muscle and intestine for transportation and absorption of nutrition. In addition, the present study has demonstrated that administration of 70 mg/kg CC had a greater influence on muscle fiber characteristics and amino acid metabolism than 140 mg/kg CC supplemented in finishing pigs. This might be relative to the fact that continuous addition of a high dose of CC can cause gastrointestinal ulcer [38].

## CONCLUSION

Dietary coated cysteamine increased the fiber density, up-regulated the expression levels of MyHC1 and MyHC2x and down-regulated expression of MyHC2b. In addition, coated cysteamine supplementation improved the deposition of amino acids by promoting amino acid transporters expression in the LDM. Above all this study presented the most appropriate dose of dietary 70 mg/kg CC supplementation, which will be useful in livestock industry to improve meat quality and nutritive value. Importantly, this study adds to the knowledge of cysteamine function but further research about underlying mechanisms are required.

## Figures and Tables

**Table 1:** Composition and calculated nutrient levels of the basal diet (air-dry basis)

Items	Content
Ingredients (g/kg)	
Corn (4.52% crude protein)	580.0
Soybean meal (8.84%)	200.0
Wheat bran	80.0
Rice bran	100.0
Vitamin premix1)	20.0
Mineral premix1)	20.0
Total	1000
Nutrient content2)	
Digestible energy (MJ/kg)	13.12
Crude protein (%)	16.40
Calcium (%)	0.80
Total phosphorus (%)	0.60
Available phosphorus (%)	0.40
Lysine (%)	0.77
Methionine (%)	0.32
Methionine+cysteine (%)	0.60

1) Provided per kilogram of diet: vitamin A, 80,000 IU; vitamin D_3_, 20,000 IU; vitamin E, 300 mg; vitamin K, 30 mg; vitamin B_1_, 30 mg; vitamin B_2_, 60 mg; vitamin B_6_, 30 mg; biotin, 0.2 mg; folic acid, 10 mg; niacin, 300 mg; pantothenic acid, 300 mg; Cu (CuSO_4_**.**5H_2_O), 12 mg; Fe (FeSO_4_**.**7H_2_O), 150 mg; Mn (MnSO_4_**.**H_2_O), 5 mg; Se (NaSeO_3_), 0.45 mg; Zn (ZnO), 150 mg.

2) Based on the composition of ingredients provided by the NRC [11].

**Table 2:** Primers used for quantitative real-time polymerase chain reaction

Gene	Accession No.	Primer, 5′–3′	Size (bp)	T_A_ (°C)
*GAPDH*	NM_001206359.1	F: ACTCACTCTTCTACCTTTGATGCT		
		R: TGTTGCTGTAGCCAAATTCA	100	59
*MyHC1*	NM_001190422	F: AAGGGCTTGAACGAGGAGTAGA		
		R: TTATTCTGCTTCCTCCAAAGGG	114	57
*MyHC2a*	NM_214136.1	F: GCTGAGCGAGCTGAAATCC		
		R: ACTGAGACACCAGAGCTTCT	137	58
*MyHC2b*	NM_001123141.1	F: ATGAAGAGGAACCACATTA		
		R: TTATTGCCTCAGTAGCTTG	166	52
*MyHC2x*	NM_001104951.1	F: AGAAGATCAACTGAGTGAACT		
		R: AGAGCTGAGAAACTAACGTG	149	55
*ASCT2*	XM_003127238.1	F: GATTGTGGAGATGGAGGATGTGG		
		R: TGCGAGTGAAGAGGAAGTAGATGA	128	60
*CAT1*	NM_001012613.1	F: TCTGGTCCTGGGCTTCATAA		
		R: ACCTTCGTGGCATTGTTCAG	123	58
*CAT2*	NM_001110420.1	F: ACAACTGGCGAAGAAGTCCG		
		R: CTGCCGAGACCCCAAAATAG	100	60
*y*^+^*LAT1*	XM_013978228.1	F: GAGTGCCAGAACACAAACGA		
		R: TCCTCCATCTTCCAAATCCA	116	58
*LAT2*	XM_011978238.1	F: CACCATTCCCTGGCTACTCT		
		R: TCCTACCACTGCCTGACAAA	185	58
*b*^0,+^*AT*	NM_001110171.1	F: GCCTATCAAGGTGCCCATC		
		R: AGCGGACGAACAGGAAGTAA	155	59

T_A_, annealing temperature; *GAPDH*, glyceraldehyde-3-phosphate dehydrogenase; *MyHC*, myosin heavy chain; *ASCT2*, autologous stem cell transplantion 2; *CAT*, chloramphenicol acetyltransferase; *y+LAT1*, y^+^-L-type amino acid transporter 1; *LAT2*, L-type amino acid transporter 2; *b0,+AT*, b^0,+^ amino acid transporter.

**Table 3:** Effects of dietary coated cysteamine hydrochloride (CC) on plasma biochemical indices in finishing pigs (n = 8)

Items	Dietary level of CC1)			p-value

	CON	CC70	CC140	
Total protein (mmol/L)	69.84±2.34	72.20±1.74	72.78±2.74	0.644
Triglyceride (mmol/L)	0.22±0.15	0.17±0.09	0.21±0.09	0.593
Total cholesterol (mmol/L)	2.18±0.07^b^	1.86±0.09^a^	2.09±0.04^b^	0.015
Glucose (mmol/L)	4.69±0.10	4.86±0.15	5.23±0.23	0.097
Urea (mmol/L)	7.47±0.37	7.73±0.47	6.62±0.67	0.313
Alkaline phosphatase (U/L)	142.45±11.96	124.55±8.85	113.76±10.91	0.174
Alanine aminotransferase (U/L)	50.76±1.80^ab^	52.54±2.77^b^	44.04±2.47^a^	0.048
Aspartate aminotransferase (U/L)	38.15±2.43	46.86±3.66	40.15±11.98	0.211

1) Con, finishing pigs fed a basal diet containing 0 mg/kg coated cysteamine hydrochloride; CC70, finishing pigs fed a basal diet containing 70 mg/kg coated cysteamine hydrochloride; CC140, finishing pigs fed a basal diet containing 140 mg/kg coated cysteamine hydrochloride.

a,b Mean values with unlike letters were significantly different (p<0.05).

**Table 4:** Effect of dietary coated cysteamine hydrochloride (CC) on muscle fiber characteristics (n = 8)

Items2)	Dietary level of CC1)			p-value

	CON	CC70	CC140	
Fiber diameter (μm)	59.1±0.91	56.67±1.83	59.54±0.96	0.264
Fiber density (n/μm^2^)	241.17±12.30^a^	284.45±9.86^b^	249.04±9.69^a^	0.013

1) CON, finishing pigs fed a basal diet containing 0 mg/kg coated cysteamine hydrochloride; CC70, finishing pigs fed a basal diet containing 70 mg/kg coated cysteamine hydrochloride; CC140, finishing pigs fed a basal diet containing 140 mg/kg coated cysteamine hydrochloride.

2) Muscle fiber diameter and density were calculated by total number of fibers and cross-sectional area of fibers.

a,b Mean values with unlike letters were significantly different (p<0.05).

**Table 5:** Effects of dietary coated cysteamine hydrochloride (CC) on amino acid composition in the longissimus dorsi muscle of finishing pigs (n = 8)

Items (mg/kg)	Dietary level of CC1)			p-value

	CON	CC70	CC140	
Essential amino acids				
Met	43.93±4.03	54.27±3.11	42.68±4.91	0.113
Arg	100.41±25.7	122.48±8.27	96.68±4.32	0.471
Ile	91.72±4.52^a^	108.21±4.54^b^	88.56±6.79^a^	0.038
Leu	152.36±9.16	178.4±9.6	149.14±10.66	0.093
Trp	24.40±1.62	27.28±2.56	23.24±1.81	0.371
Phe	120.98±5.91^ab^	139.57±7.43^b^	114.38±6.51^a^	0.037
Lys	141.83±11.9	145.96±9.56	115.89±5.07	0.067
His	79.34±6.05^a^	101.43±5.39^b^	84.34±6.67^ab^	0.044
Val	99.02±4.94	115.38±8.69	95.33±9.12	0.179
Thr	94.46±7.96	107.22±5.93	85.91±5.88	0.099
Eessential amino acid (total)	948.45±68.33	1,100.20±52.16	893.15±54.04	0.050
Non-essential amino acids				
Glu	117.69±16.83	166.67±30.85	123.63±20.82	0.298
Ser	95.38±11.83	110.36±7.85	85.04±5.36	0.146
Ans	678.20±38.29^a^	846.55±37.21^b^	698.99±60.84^a^	0.037
Asp	45.46±4.98	48.6±4.83	34.39±5.38	0.139
Ala	565.75±26.52	660.38±58.51	503.06±39.63	0.057
Gly	267.91±23.89	259.49±11.28	229.29±28.26	0.451
Cys	12.76±1.43	12.82±1.54	10.70±2.16	0.724
Pro	67.40±21.54	108.38±9.34	86.95±4.62	0.136
Tyr	95.90±4.78	103.74±4.39	86.14±5.43	0.059
Sar	56.36±3.84	52.76±3.56	51.33±5.23	0.696
Total amino acid	2,951.26±148.16^ab^	3,469.95±168.89^b^	2,805.65±207.27^a^	0.036

1) CON, finishing pigs fed a basal diet containing 0 mg/kg coated cysteamine hydrochloride; CC70, finishing pigs fed a basal diet containing 70 mg/kg coated cysteamine hydrochloride; CC140, finishing pigs fed a basal diet containing 140 mg/kg coated cysteamine hydrochloride.

a,b Mean values with unlike letters were significantly different (p<0.05).

## References

[1] Grunert KG, Bredahl L, Brunsø K (2004). Consumer perception of meat quality and implications for product development in the meat sector—a review. Meat Sci.

[2] Lefaucheur L, Milan D, Ecolan P (2004). Myosin heavy chain composition of different skeletal muscles in large white and meishan pigs. J Anim Sci.

[3] Joo ST, Kim GD, Hwang YH (2013). Control of fresh meat quality through manipulation of muscle fiber characteristics. Meat Sci.

[4] Lee S, Joo S, Ryu Y (2010). Skeletal muscle fiber type and myofibrillar proteins in relation to meat quality. Meat Sci.

[5] Kim GD, Yang HS, Jeong JY (2018). Intramuscular variations of proteome and muscle fiber type distribution in *semimembranosus* and *semitendinosus* muscles associated with pork quality. Food Chem.

[6] Zhou P, Zhang L, Li J (2015). Effects of dietary crude protein levels and cysteamine supplementation on protein synthetic and degradative signaling in skeletal muscle of finishing pigs. Plos One.

[7] Zhang ZY, Yang MF, Wang T (2015). Cysteamine alleviates early brain injury via reducing oxidative stress and apoptosis in a rat experimental subarachnoid hemorrhage model. Cell Mol Neurobiol.

[8] Bai MM, Liu HN, Xu K (2017). Effects of dietary coated cysteamine hydrochloride on pork color in finishing pigs. J Sci Food Agric.

[9] Liu G, Wang Z, Wu D (2009). Effects of dietary cysteamine supplementation on growth performance and whole-body protein turnover in finishing pigs. Livest Sci.

[10] Zhou P, Luo Y, Zhang L (2017). Effects of cysteamine supplementation on the intestinal expression of amino acid and peptide transporters and intestinal health in finishing pigs. Anim Sci J.

[11] NRC (2012). Nutrient requirements of swine.

[12] Deng J, Wu X, Bin S (2010). Dietary amylose and amylopectin ratio and resistant starch content affects plasma glucose, lactic acid, hormone levels and protein synthesis in splanchnic tissues. J Anim Physiol Anim N.

[13] Turek Z, Grandtner M, Kreuzer F (1972). Cardiac hypertrophy, capillary and muscle fiber density, muscle fiber diameter, capillary radius and diffusion distance in the myocardium of growing rats adapted to a simulated altitude of 3500 m. Pflügers Archiv.

[14] Qin PY, Ding YF, Xiao JH (2017). Effects of a traditional Chinese medicine formula and its extraction on muscle fiber characteristics in finishing pigs, porcine cell proliferation and isoforms of myosin heavy chain gene expression in myocytes. Asian-Australas J Anim.

[15] Hu CJ, Jiang QY, Zhang T (2017). Dietary supplementation with arginine and glutamic acid modifies growth performance, carcass traits, and meat quality in growing-finishing pigs. J Anim Sci.

[16] Tan B, Yin Y, Liu Z (2011). Dietary l-arginine supplementation differentially regulates expression of lipid-metabolic genes in porcine adipose tissue and skeletal muscle. J Nutr Biochem.

[17] Zhang C, Luo J, Yu B (2015). Dietary resveratrol supplementation improves meat quality of finishing pigs through changing muscle fiber characteristics and antioxidative status. Meat Sci.

[18] Yang CB, Li AK, Yin YL (2005). Effects of dietary supplementation of cysteamine on growth performance, carcass quality, serum hormones and gastric ulcer in finishing pigs. J Sci Food Agric.

[19] Barnett M, Hegarty R (2016). Cysteamine: a human health dietary additive with potential to improve livestock growth rate and efficiency. Anim Prod Sci.

[20] Bai MM, Liu HN, Xu K (2018). Effects of dietary coated cysteamine hydrochloride on pork color in finishing pigs. J Sci Food Agric.

[21] Yu Q, Wu W, Tian X (2017). Unraveling proteome changes of holstein beef m. Semitendinosus and its relationship to meat discoloration during post-mortem storage analyzed by label-free mass spectrometry. J Proteomics.

[22] Ryu YC, Lee MH, Lee SK (2006). Effects of muscle mass and fiber type composition of *longissimus dorsi* muscle on postmortem metabolic rate and meat quality in pigs. J Muscle Foods.

[23] Jeong DW, Choi YM, Lee SH (2010). Correlations of trained panel sensory values of cooked pork with fatty acid composition, muscle fiber type, and pork quality characteristics in Berkshire pigs. Meat Sci.

[24] Gondret F, Lefaucheur L, Juin H (2006). Low birth weight is associated with enlarged muscle fiber area and impaired meat tenderness of the longissimus muscle in pigs. J Anim Sci.

[25] Schiaffino S, Reggiani C (1996). Molecular diversity of myofibrillar proteins: gene regulation and functional significance. Physiol Rev.

[26] Kim GD, Jeong JY, Hur SJ (2010). The relationship between meat color (CIE L* and a*), myoglobin content, and their influence on muscle fiber characteristics and pork quality. Korean J Food Sci Anim Resour.

[27] Cleveland BD, Bower CG, Redfield AL (2015). Effect of feeding distiller’s grains and supplementing with dietary antioxidants on ground beef color during retail display. Meat Sci.

[28] Korn KT, Lemenager RP, Claeys MC (2013). Supplemental vitamin D3 and zilpaterol hydrochloride. II. Effect on calcium concentration, muscle fiber type, and calpain gene expression of feedlot steers. J Anim Sci.

[29] Hu R, Wang Z, Peng Q (2016). Effects of ghrp-2 and cysteamine administration on growth performance, somatotropic axis hormone and muscle protein deposition in yaks (*Bos grunniens*) with growth retardation. Plos One.

[30] Kamalakar RB, Chiba LI, Divakala KC (2009). Effect of the degree and duration of early dietary amino acid restrictions on subsequent and overall pig performance and physical and sensory characteristics of pork. J Anim Sci.

[31] Liu G, Wei Y, Wang Z (2008). Effects of dietary supplementation with cysteamine on growth hormone receptor and insulin-like growth factor system in finishing pigs. J Agric Food Chem.

[32] Zhang S, Qiao S, Ren M (2013). Supplementation with branched-chain amino acids to a low-protein diet regulates intestinal expression of amino acid and peptide transporters in weanling pigs. Amino Acids.

[33] Laspiur JP, Burton JL, Weber PSD (2009). Dietary protein intake and stage of lactation differentially modulate amino acid transporter mrna abundance in porcine mammary tissue. J Nutr.

[34] Wu G, Bazer FW, Satterfield MC (2013). Impacts of arginine nutrition on embryonic and fetal development in mammals. Amino Acids.

[35] He L, Yang H, Hou Y (2013). Effects of dietary L-lysine intake on the intestinal mucosa and expression of cat genes in weaned piglets. Amino Acids.

[36] Fuchs BC, Bode BP (2005). Amino acid transporters asct2 and lat1 in cancer: partners in crime?. Semin Cancer Biol.

[37] Verrey F, Closs EI, Wagner CA (2004). CATs and HATs: the SLC7 family of amino acid transporters. Pflügers Arch.

[38] Mcleod KR, Harmon DL, Schillo KK (1995). Cysteamine-induced depletion of somatostatin in sheep: time course of depletion and changes in plasma metabolites, insulin, and growth hormone. J Anim Sci.

